# Usability testing of ANSWER: a web-based methotrexate decision aid for patients with rheumatoid arthritis

**DOI:** 10.1186/1472-6947-13-131

**Published:** 2013-12-01

**Authors:** Linda C Li, Paul M Adam, Anne F Townsend, Diane Lacaille, Charlene Yousefi, Dawn Stacey, Diane Gromala, Chris D Shaw, Peter Tugwell, Catherine L Backman

**Affiliations:** 1 Department of Physical Therapy, University of British Columbia, Vancouver, Canada; 2 Arthritis Research Centre of Canada, Vancouver, Canada; 3 Mary Pack Arthritis Program, Vancouver Coastal Health, Vancouver, Canada; 4 Division of Rheumatology, Faculty of Medicine, University of British Columbia, Vancouver, Canada; 5 School of Nursing, University of Ottawa, Ottawa, Canada; 6 Ottawa Hospital Research Institute, Ottawa, Canada; 7 School of Interactive Arts and Technology, Simon Fraser University, Surrey, Canada; 8 Institute of Population Health, University of Ottawa, Ottawa, Canada; 9 Department of Occupational Science and Occupational Therapy, University of British Columbia, Vancouver, Canada

**Keywords:** Patient decision aid, Rheumatoid arthritis, Methotrexate, Usability test

## Abstract

**Background:**

Decision aids are evidence-based tools designed to inform people of the potential benefit and harm of treatment options, clarify their preferences and provide a shared decision-making structure for discussion at a clinic visit. For patients with rheumatoid arthritis (RA) who are considering methotrexate, we have developed a web-based patient decision aid called the ANSWER (Animated, Self-serve, Web-based Research Tool). This study aimed to: 1) assess the usability of the ANSWER prototype; 2) identify strengths and limitations of the ANSWER from the patient’s perspective.

**Methods:**

The ANSWER prototype consisted of: 1) six animated patient stories and narrated information on the evidence of methotrexate for RA; 2) interactive questionnaires to clarify patients’ treatment preferences. Eligible participants for the usability test were patients with RA who had been prescribed methotrexate. They were asked to verbalize their thoughts (i.e., think aloud) while using the ANSWER, and to complete the System Usability Scale (SUS) to assess overall usability (range = 0-100; higher = more user friendly). Participants were audiotaped and observed, and field notes were taken. The testing continued until no new modifiable issues were found. We used descriptive statistics to summarize participant characteristics and the SUS scores. Content analysis was used to identified usability issues and navigation problems.

**Results:**

15 patients participated in the usability testing. The majority were aged 50 or over and were university/college graduates (n = 8, 53.4%). On average they took 56 minutes (SD = 34.8) to complete the tool. The mean SUS score was 81.2 (SD = 13.5). Content analysis of audiotapes and field notes revealed four categories of modifiable usability issues: 1) information delivery (i.e., clarity of the information and presentation style); 2) navigation control (i.e., difficulties in recognizing and using the navigation control buttons); 3) layout (i.e., position of the videos, text, diagrams and navigation buttons); 4) aesthetic (i.e., the colour, look and feel of the online tool).

**Conclusions:**

Although the SUS score indicated high usability before and after major modification, findings from the think-aloud sessions illustrated areas that required further refinement. Our results highlight the importance of formative evaluation in usability testing.

## Background

Rheumatoid arthritis (RA) affects about 1% of the population worldwide, with the peak onset between age 35 and 50 [1,2]. There is ample evidence supporting early and persistent use of disease-modifying anti-rheumatic drugs (DMARD) to prevent irreversible joint damage [3-5]. Among the available DMARD, methotrexate is generally considered the first-line treatment for RA based on its benefits and potential side effects. However, a Canadian population-based research reported that only 43% of the population with RA had used a DMARD over a five-year period [6].

Patients’ decisions on medication use can be affected by their concerns about side effects [7]. Several qualitative studies in chronic disease, including RA, have revealed patients’ ambivalence toward using medication [8,9]. On one hand, they described an aversion to drugs because of the anticipated side effects and, on the other hand, they felt compelled to take medication due to a fear of the potentially crippling effects of an uncontrolled disease. The circumstance in which people decided to use or not use medications appeared to be influenced by the nature of the symptoms and the extent to which symptoms disrupt daily lives.

In recent years, clinical practice has been expanding from traditional authoritative models, in which physicians make treatment decisions for patients, to include shared decision-making. This involves an exchange of information to prepare patients to make treatment decisions and engage in the process of decision-making with their healthcare providers [10,11]. One way to facilitate shared decision-making is through the use of patient decision aids [12]. These are evidence-based tools designed to help individuals to choose between two or more treatment options [13,14]. Decision aids help people to personalize information about treatment effectiveness, outcomes and the inherent uncertainties of potential benefit versus potential harm. An important feature of decision aids is that they help individuals to clarify their personal values towards benefits and harms, and to communicate this information to health professionals. Patients who have used decision aids tend to have more knowledge about the treatment, more realistic expectations and lower decisional conflict compared to those who received usual care [15]. Also, decision aid users are more likely to participate in decision-making and to reach a treatment decision [15].

To assist patients with RA to make decisions about using methotrexate, we developed an online decision aid called ANSWER (Animated, Self-serve, Web-based Research Tool) [16]. The innovative aspect of ANSWER is its built-in patient stories that illustrate common situations people experience when making decisions about their treatment, as well as attributes required for effective management of their healthcare. The primary objective of this study was to assess the user friendliness of the ANSWER prototype. Our secondary objective was to identify strengths and limitations of the tool from the user’s perspective. This study focuses on the refinement of the ANSWER prototype so that it could be deployed for use by the general public.

## Methods

### Decision aid development

Development of the ANSWER decision aid was guided by the International Patient Decision Aid Standards [17,18]. Our target users were individuals who had been prescribed methotrexate for RA, but were feeling unsure about starting it. As methotrexate was usually prescribed at the early stage of RA, we designed the ANSWER with the needs of newly diagnosed patients in mind. This decision aid focused on two options: 1) to take methotrexate as prescribed; 2) to refuse methotrexate and talk to the doctor about other treatment options. The design of the ANSWER was guided by Jibaja-Weiss’s Edutainment Decision Aid Model [19]. Educative entertainment, or edutainment, is a process whereby educational messages are imbedded within an entertaining medium, such as broadcasting media, e.g., television [20,21] or performing arts (e.g., theatre) [22], and games [22-25]. Central to the Edutainment Decision Aid Model is the focus on making the computer-human interface user-friendly [19,26]. We assembled a multidisciplinary team, involving patients/consumers, digital media experts, clinicians and health researchers, to develop the online tool. The role of patients/consumers was particularly important as they had firsthand experience in making treatment decisions. They informed the design of the ANSWER by sharing their experiences of using computers while having joint pain and fatigue. Further, they reviewed the content of the patient decision aid to ensure it is understandable by people without medical background, although no readability program was applied.

**Figure 1:**
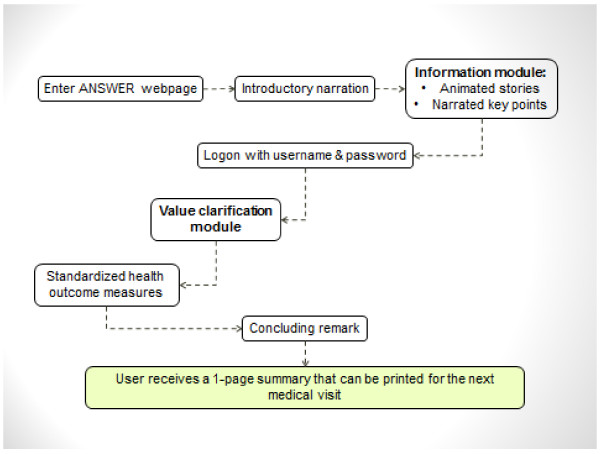
ANSWER navigation pathway*. *The ANSWER is designed to guide patients to navigate each component in sequence. The dash arrows indicate that patients may also access any component without following a linear path.

Figure 1 presents the navigation path of the ANSWER. Users were guided to start by completing the Information Module, the Value Clarification Module, and then the standardized health outcome measures. However, the tool also allowed users to access any component without following a linear path. The *Information Module* consisted of the latest evidence on methotrexate compared to placebo from a Cochrane systematic review [27] and the current evidence-based recommendations from the 3E (Evidence, Expertise, Exchange) Initiative [28]. The latter was a multinational collaboration involving 751 rheumatologists from 17 countries to develop recommendations for the use of methotrexate in RA using a Delphi process. The design of ANSWER was guided by our previous qualitative study on the help-seeking experience of patients with early RA [29], and input from the patient/consumer collaborators. The module addressed six topics: 1) About RA; 2) About methotrexate; 3) Side effects of methotrexate; 4) Pregnancy; 5) Alcohol use; 6) Other medication options and adjunctive treatments (e.g., exercise, joint protection techniques). Recognizing that patients had different preferences in receiving information, the information was provided in text, voice narration and animated vignettes.

**Figure 2:**
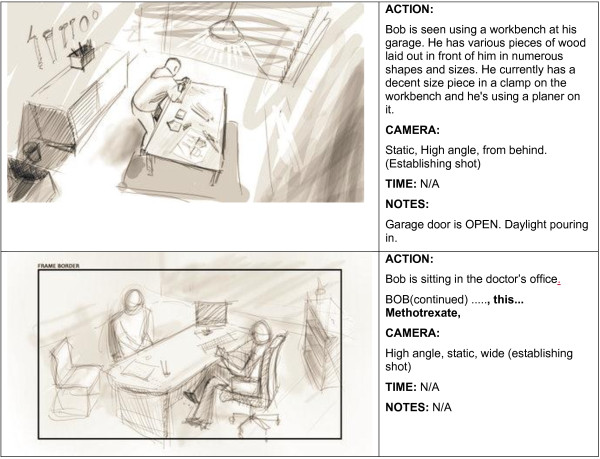
Sample storyboard for ‘About Methotrexate – Bob’s Story’.

Each of the six vignettes was based on a unique, fictional character (Figure 2: Sample Storyboard). We used the animated graphic novel approach for the animated component, which is a relatively simple, inexpensive and visually appealing method for creating the animation sequences. This involved repeatedly photographing real actors in key poses based on the story script, processing the images in Photoshop to create a comic-book-inspired look, and then sequencing the images to create a limited key-frame animation for the characters. This animation method also allows us to make modification to characters’ appearances, so that they appear to be ‘race neutral’ for a multinational audience in Canada. We used a slow animated sequence of 3–5 frames/10 seconds, which allowed us the freedom to emphasize the more important points in the story. Finally, actor voice-over was added to complete the animated vignettes.

In the *Value Clarification Module*, two methods were used to assist patients to consider the importance of the consequences from each option. First, they were asked to rate on a 5-point scale the importance of: 1) improving joint pain; 2) preventing joint damage; 3) improving physical function; 4) avoiding side effects; 5) becoming pregnant /starting a family; 6) drinking alcohol. This was followed by the second method, in which they indicated the relative importance by allocating 100 points across the same six items. Patients were also asked to list their questions and concerns about using methotrexate and to indicate their preferred choice out of the two options, or to declare that they remained uncertain.

The ANSWER tool ends with two standardized health status questionnaires: the Health Assessment Questionnaire [30] and the RA Disease Activity Index [31,32]. Scores of these measures and the individual’s response to the value clarification questions were summarized in a 1-page printable report at the end of the online program. Patients could discuss this report with their physicians before reaching a final decision about using methotrexate. The ANSWER prototype was reviewed by the research team, patients/consumers (OK, CK, CM) and a health education consultant (GE) to ensure that the content was understandable to people without a research or clinical background.

### Usability testing

Guided by the methods outlined by Rubin and Chisnell [33], we used an iterative testing protocol, whereby we 1) conducted onsite testing with participants to identify usability issues in the ANSWER prototype, 2) stopped testing and made modifications when no new issues were identified, and 3) resumed testing with the modified version. A usability issue was defined as 1) when a participant was not able to advance to the next step due to the decision aid design or a programming error, or 2) when a participant was distracted by a particular design or content of the online tool. Prior to the testing, we recognized that some usability issues would not be modifiable. For example, because the animated stories were in their final format, we were unable to change the animation style or the storylines. We continued the testing until no modifiable usability issues were identified.

Participants were recruited through study flyers posted at 1) rheumatologists’ offices and community health centres in Vancouver, 2) Mary Pack Arthritis Program, Vancouver General Hospital and 3) classified advertisement websites such as Craigslist and Kijiji. Eligible individuals were patients who had a diagnosis of RA and had been prescribed methotrexate. After providing written informed consent, participants attended a two-hour testing session at the Arthritis Research Centre of Canada. The test was conducted in a small meeting room in the presence of a trained research staff member. Participants were instructed to use the ANSWER as if they were looking for information about methotrexate for RA. We used the concurrent think-aloud approach. The think-aloud protocol was developed in its current form by Ericsson and Simon [34], and was introduced to the field of human-computer interaction by Lewis [35]. Participants were encouraged to verbalize thoughts and feelings when navigating the decision aid. The research staff prompted the participant to elaborate on his/her comments when appropriate or when they fell silent for a while. For example, participants were asked, “What are you thinking?” or “Can you describe what are looking at”, if they fell silent. In addition, the research staff intervened when participants indicated they did not know how to progress to the next stage while using the ANSWER. All sessions were audio-recorded. To capture situations which might be missed by the audio recording, the research staff took detailed field notes throughout the session.

Participants were then asked to complete a questionnaire including the System Usability Scale (SUS) [36] and socio-demographic and internet use characteristics. Developed by Brooke [36], the SUS consists of 10 statements that are scored on a 5-point scale of strength of agreement. The total ranges from 0 to 100, with a higher score indicating more user-friendly. Originally developed to measured system usability, the SUS has been adapted for testing a wide range of technologies, including hardware platforms and software programs [37].

### Data analysis

We used descriptive statistics to summarize participant characteristics and the SUS score after each testing cycle. No statistical comparisons were conducted between cycles, as hypothesis testing was not a goal of this study. Audio-recordings were transcribed verbatim. Content analysis was conducted to identify 1) modifiable usability issues and navigation problems, and 2) strength and limitation of the ANSWER design. Our data analysis was inductive, as we sought to understand participants’ experience with the ANSWER rather than to prove a preconceived theory. We used a constant comparisons approach, whereby participants’ experiences in using the ANSWER were coded. Codes that reveal similar navigation problems were grouped into categories [38]. The data were constantly revisited after the initial coding, until it was clear no new categories emerged. The coding process was performed by one researcher (LCL) who read each transcript and attributed a code to sentences or paragraphs (open coding). Other team members were also included in the coding process to assess causes of usability problems from participants’ comments. Axial coding was performed to develop connections among the categories of usability problems. LCL was also responsible for discussing the modifications required with the software programmer, and supervised the revisions. We stopped a testing cycle to make modifications when no new problem was identified. The study protocol was approved by the University of British Columbia Behavioural Research Ethics Board (Application number: H09-00898).

## Results

We recruited 15 eligible participants between August and October 2010. Of those, 10 participated in Cycle 1 and five tested the revised version in Cycle 2 (Table 1). We did not identify any new issues in Cycle 2. Over half of the participants were aged 50 or older, with 85.7% being women and 53.3% being university or college graduates. The median disease duration was 5 years (interquartile range [IQR]: 0.83; 10.00), with participants in Cycle 1 having a longer median disease duration (5.50 years [IQR: 0.65; 11:00] versus 2 years [IRQ: 0.92; 15.50]). All participants have used methotrexate. They all used the internet for emails and 46.7% used it to play internet games. Participants took an average of 56.80 minutes (SD = 34.80) to complete the ANSWER. Table 2 presents the total SUS score and the results of individual items in Cycles 1 and 2. The SUS scores were similar before and after modification of the online tool (Cycle 1: 81.25, SD = 14.92; Cycle 2: 81.00, SD = 11.81).

**Table 1:** Participant characteristics and experience with internet

	**All (n = 15)**	**Cycle 1: Before modification (n = 10)**	**Cycle 2: After modification (n = 5)**
**Age**			
**20–34**	2 (13.3%)	1 (10.0%)	1 (20.0%)
**35–49**	5 (33.3%)	4 (40.0%)	1 (20.0%)
**50–64**	7 (46.7%)	4 (40.0%)	3 (60.0%)
**65 or older**	1 (6.7%)	1 (10.0%)	0
**Women**	13 (85.7%)	9 (90.0%)	4 (80.0%)
**University/college graduates**	8 (53.3%)	6 (60.0%)	2 (40.0%)
**Disease duration in Years – Median (IQR)**	5.00 (0.83; 10.00)	5.50 (0.65; 11.00)	2.00 (0.92; 15.50)
**Hours spent on Internet per day - Median (IQR)**	2.00 (1.00; 3.00)	1.75 (1.00; 2.25)	2.50 (0.88; 3.75)
**Use of internet for:**			
**Email**	15 (100.0%)	10 (100.0%)	5 (100.0%)
**Reading news**	4 (26.7%)	3 (30.0%)	1 (20.0%)
**Entertainment**	1 (6.7%)	1 (10.0%)	0
**Gaming**	7 (46.7%)	5 (50.0%)	2 (40.0%)

*IQR* Interquartile range.

**Table 2:** ANSWER usability testing results

	**All (n = 15)**	**Cycle 1: Before modification (n = 10)**	**Cycle 2: After modification (n = 5)**
**Time to complete ANSWER in minutes (SD)**	56.08 (34.80)	55.50 (37.98)	57.00 (33.28)
**Modified system usability scale items (SD)**			
(1 = Strongly disagree; 5 = Strongly agree)**:**			
**1.** I liked using ANSWER as a tool for making an informed decision about using methotrexate as a treatment option for my RA	4.13 (1.06)	4.20 (1.23)	4.00 (0.71)
**2.** I found ANSWER unnecessarily complex	1.20 (0.56)	1.10 (0.32)	1.40 (0.89)
**3.** I thought ANSWER was easy to use	4.07 (1.34)	3.80 (1.55)	4.60 (0.55)
**4.** I think I would need the support of a technical person to be able to use ANSWER	1.60 (1.06)	1.70 (1.25)	1.40 (0.55)
**5.** I found the content and navigation in ANSWER was well integrated	3.73 (1.10)	3.60 (1.27)	4.00 (0.71)
**6.** I thought there was too much inconsistency between the design and navigation of ANSWER	1.87 (1.19)	1.60 (0.84)	2.40 (1.67)
**7.** I would imagine that most patients with RA would learn to use ANSWER very quickly	4.47 (1.06)	4.50 (1.27)	4.40 (0.55)
**8.** I found ANSWER very cumbersome to use	1.73 (1.39)	1.50 (1.27)	2.20 (1.64)
**9.** I would be very confident using ANSWER	4.13 (1.19)	4.00 (1.41)	4.40 (0.55)
**10.** I would need to learn a lot of things about using computers before I could get going with ANSWER	1.67 (0.90)	1.70 (1.06)	1.60 (0.55)
**Total System Usability Scale score (SD)**	81.17 (13.53)	81.25 (14.92)	81.00 (11.81)
(Scores of the 10 items were transformed into a summary score ranging from 0 to 100; higher = more user friendly)			

### Modifiable usability issues and changes made

**Figure 3:**
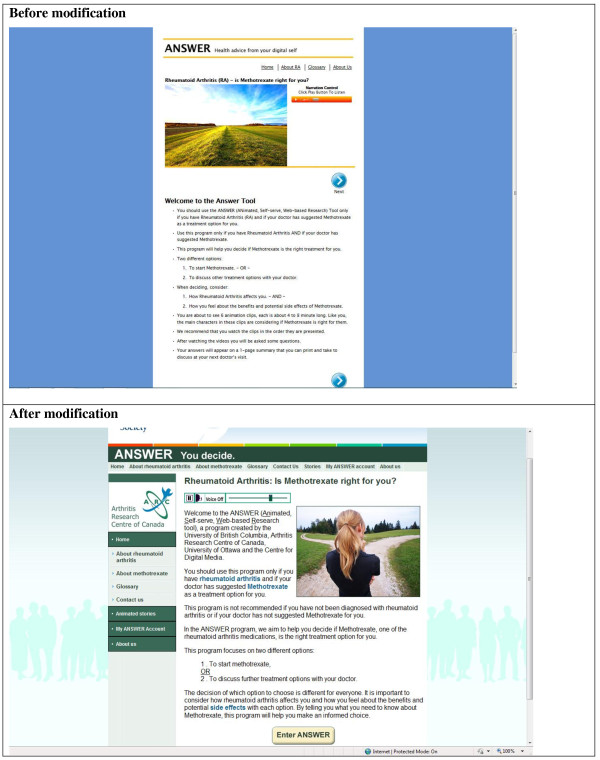
ANSWER homepage – before and after modification.

Four categories of modifiable usability issues were identified during Cycle 1 (Table 3, with examples of participants’ comments). These include 1) Information Delivery, 2) Navigation Control, 3) Layout, and 4) Aesthetic. Figure 3 presents the screenshots of the ANSWER homepage before and after modification.

**Table 3:** Modifiable usability issues identified by participants in testing cycle 1 and changes made

**Category**	**Examples of participant comment**	**Changes made**
**1. Information Delivery:** clarity of the information and presentation style.	• The narration is a bit long…a little bit repetitive. (Jamie – female, age group: 35–49)	Added key messages for users who prefer a summary of the narrated content.
	• Six video clips. That’s quite a lot especially eight minutes long. (Theresa – female, age group: 50–64)	• Reduced the length of videos. The final version ranged from 4 minutes 26 seconds to 7 minutes 55 seconds.
	• **(On videos)** I think it’s a bit long winded. You could have said the same thing with about 2 minutes less so that’s like saying a bit boring for someone to watch him doing the same thing twice or three times. (Sherly – female, age group: 50–64)	• Added subtitles to highlight important points in the video.
**2. Navigation Control:** Difficulties in recognizing and using buttons to start/stop the narrated content, adjust volume, and control videos.	• This could be a different colour maybe, the narration (*button*), just because I didn’t see it right away, I went straight to the text to read. (Bob – male, age group: 35–49)	• Used the YouTube format for all videos.
		• Enlarged the size of buttons.
	• **(On accessing the videos)** Well I’d be curious so what I would do is I would probably click on, my first inclination is to click this because, you know, you are programmed by YouTube to do that. I saw the narration button later and that’s why I was like, oh, okay, now what do I do? (Bob – male, age group: 35–49)	• Added labels to navigation controls when appropriate.
**3. Layout:** Positions of the video, text, diagrams and navigation buttons.	• **(A comment on watching video and reading information at the same time)** …I lose the video so I am like back and forth, back and forth. Keeps me busy, keeps me entertained, but not all (*the time*), you know, especially when you are dealing with people with arthritis before medication, your hands are not just scrolling down, trust me, it’s very, very hard. (Jamie – female, age group: 35–49)	• Further condensed the key points in order to reduce scrolling with a mouse while viewing a webpage.
		• Revised the webpage layout and added hyperlinks for easy access to key summaries and video.
**4. Aesthetic:** The colour and ‘look and feel’ of the program.	• For aesthetics it might be nice to have a coloured box around each one of these (*diagrams*)…I don’t know if you can make them all the same or each one different colours because it (*the website*) looks kind of bland…Or it doesn’t look like a great beginning where none of these really jump out at me… (Bob – male, age group: 35–49)	• Added pictures in the introductory pages and throughout the value elicitation module.
		Added a screenshot of the animated story at the top of each page of the information module. A hyperlink was set up to direct people to see the video in a bigger YouTube viewer.
	• As to the colour and layout, I think it needs, it’s kind of flat and uninteresting… (Theresa – female, age group: 50–64)	
	• Probably add a little more just colour. Make it a little more fun so you can actually like you are eager to go into the site. (Jamie – female, age group: 35–49)	

### Information delivery

All participants commented on the length of the ANSWER tool. The original version included details of benefits and risks of methotrexate with each video lasting 6–8 minutes long. During the testing, participants commented on the repetitiveness of the information and the video length. In light of these comments, we added short key messages throughout the online tool, reduced the video length, and included subtitles in the videos to highlight important points. It should be noted that the videos were shortened by condensing the storyline, not the evidence. The rheumatologist investigators in this team (Lacaille, Tugwell) had ensured that the change did not compromise the presentation of evidence.

### Navigation control

Participants found it difficult to use the control buttons to access the narrated content, adjust volume and control the videos. In the original version, we created our own navigation buttons for the ANSWER with the intent to achieve a unique look. This, however, became problematic during the usability testing. While some participants did not recognize these buttons, others did not know how to operate them. One participant commented that average internet users might be more comfortable with the YouTube navigation buttons and format (Bob, Table 3). Based on the feedback, we subsequently replaced the navigation controls with the YouTube format. Further, the button size was enlarged to increase ease of use for patients with hand pain.

### Layout

In the original version, each webpage under the tab ‘Animated Stories’ started with the videos, followed by written summaries of the information. Participants found the format unfriendly to navigate, especially for people who preferred to watch the video and browse the text at the same time. One participant commented that this layout required a lot of scrolling up and down with a mouse, which was particularly difficult for people with RA as the hand joints were often affected (Jamie, Table 3). In the revised version, we further condensed the key messages to reduce scrolling with a mouse within a webpage. In addition, we added hyperlinks throughout the tool to improve access to the videos and written information.

### Aesthetic

A major criticism of the original ANSWER tool was its aesthetic. One participant commented that the colour was ‘flat and uninteresting’ (Theresa, Table 3). Another participant felt that it needed more colour to make the site ‘a little more fun’ and more inviting (Jamie). Based on the feedback, we included pictures in the introductory pages and throughout the Value Clarification Module. In addition, we added colourful screenshots from the animated stories in the Information Module.

### Limitations and strengths of the ANSWER patient decision aid

Although some components of the ANSWER were not modifiable (e.g., storylines of the animated videos), we acknowledged participants’ comments regarding limitations of this online tool. Four additional themes related to limitations and strengths of the ANSWER emerged in our analysis. These included 1) authenticity, 2) information accuracy about living with arthritis, 3) modeling shared decision making, and 4) ease of use (Table 4).

**Table 4:** Themes illustrating limitations and strengths of the ANSWER design

**Category**	**Participant comments**
**1. Authenticity:** Participants were able to relate to the patient stories, as they cover different age groups and sexes. However, some preferred real actors as compared to the animated characters.	• …it seems like real information, real people talking about the disease, pros and cons, you know, the fear to take it, uh, the fears or stopping it could happen; are they going to be able to work? The fear of losing a job. So those are real situations. It just makes the site a little more human and realistic. It’s not just scientific information. (Jamie – female, age group: 35–49; C1)
	• **(On ‘Rosa: About RA video’)** I kind of related to it just because, well…, because my daughter’s, like I did have problems, like I did start flaring up. I was in remission and then I flared up after. So I was kind of relating to her …and then hearing her (*Rosa*) talk to her father really made me sad to actually go back to my parents’ place while my husband was working and stay there for the week. It was tough, but yeah, I can relate to it. (Amy – female, age group: 20–34; C1)
	• I think the information is very plentiful, but I think what people, what the layman person to look at this website is going to need to know more personable, real stories from people that are like not acting, not – you know people that are actually taking the drug on a regular basis what they’re going through. (Rosemary – female, age group: 35–49; C1)
	• It might be good overall if these were real video clips (*with actors*). (Sheila – female, age group: 50–64; C2)
**2. Information accuracy about living with arthritis:** Key feature of RA was fairly portrayed.	• People surrounding, you know, patients, um, with RA, they don’t know like…that tiredness you feel at all times the people around you they don’t really understand. So if someone in my family or within, you know, a family watches this they might go like, oh it’s true, I mean she’s not like making it up, um, she is actually tired; it’s part of the information, so that’s something that haven’t seen in any of the websites to be honest. (Jamie – female, age group: 35–49; C1)
**3. Modeling shared decision-making:** Some participants commented on the ability of ANSWER to provide examples of active and engaged patients.	• I think we’re moving away from the old style where you just did whatever your doctor told you and didn’t ask questions. And (*ANSWER*) is helpful in expanding people’s thinking about it. As far as I can see it touched on the key decision point. So it’s good for that. (Theresa – female, age group: 50–64; C1)
**4. Ease of use:** Participants commented on the user friendliness, but they were also hoping for a more sophisticated software product	• It was user-friendly definitely. Yeah, I feel it wasn’t sophisticated enough. I mean methotrexate is a big name and it was a little gimmicky at some point (*of the video presentation*), maybe because of the graphics you know. (Jane – female, age group: 50–64; C2)

*C1* Usability Testing Cycle 1, *C2* Usability Testing Cycle 2.

*RA* Rheumatoid Arthritis.

In general, participants from both cycles were able to relate to the characters in one or more patient stories, although some preferred the stories told by real actors or patients rather than animated characters. Also, they felt that the pros and cons about using methotrexate were well integrated in the context of everyday life of people with RA. For example, one participant remarked positively about the realistic depiction of fatigue in the stories (Jamie, Table 4). Participants also commented that the patient stories were helpful because the main characters demonstrated shared decision-making behaviours, such as considering pros and cons of treatment options and communicating questions and concerns with health professionals. Finally, although participants felt in general that the ANSWER was user-friendly, some criticised the videos as less polished compared to other existing patient education programs that used real patients or actors (Table 4).

## Discussion

In this study, we employed rigorous methodology to assess the usability of a new online decision aid for patients with rheumatoid arthritis. Our results showed that the ANSWER prototype was user-friendly even before modifications were made (overall SUS score before modification: 81.25, SD = 14.92; after modification: 81.00, SD = 11.81). Component scores of the two cycles appeared to be similar, although the small sample size hindered the opportunity for hypothesis testing. There is no consensus on what constitutes an acceptable SUS score [36], however Bangor et al. [37] reviewed the measurement properties of the SUS and suggested that products with SUS scores between the high 70s and 80s were considered ‘good products’. Programs scoring below 70 required further improvement and those in the low 70s were considered ‘passable’. Products scoring 90 and above were deemed ‘superior’. Based on their recommendation, the ANSWER has met the standard of a user friendly program. It should be noted that we designed the ANSWER for patients to use at their own pace. Although participants were asked to complete the ANSWER in one testing session, we expect that in reality some might complete the online tool in several sessions.

It was expected the usability of a new product could be improved by addressing issues identified during the usability testing and that this might translate into an improved SUS. What was interesting in this study was that although the SUS score was high and met the standard as a good product in Round 1, the formative evaluation identified a number of modifiable usability issues. This supports the use of the formative evaluation along with the summative evaluation in usability testing. The small change in the SUS score between Round 1 and Round 2 might be due to the non-modifiable issues, including those raised about the videos. However, given the small sample size in each round, a direct comparison would not be possible.

Our study also demonstrated the value of formative usability testing. Despite the favourable SUS scores in Cycle 1, participants identified a number of usability issues. Our findings were similar to the usability issues found in other patient-oriented online programs. For example, Stinson et al. [39] tested an electronic chronic pain diary for adolescents with arthritis and found the slider controls of pain visual analogue scales difficult to operate. These slider controls were subsequently modified to improve user experience. In another study evaluating an online self-management program for youth with juvenile idiopathic arthritis, Stinson et al. [40] uncovered performance errors and design issues that were modifiable to improve user satisfaction. Recently, in a full scale usability evaluation of an online interactive game for patients making treatment decisions for prostate cancer, Reichlin et al. [41] identified similar navigation and content-related issues that could impede user experiences. These studies indicated the importance of formative usability testing to improve new online programs prior to field testing. Findings from the current usability testing concur with this viewpoint.

There are several limitations with this study. First, the testing was conducted with participants with a long disease duration (median = 5 years), hence the view of those with a recent diagnosis was under represented. Second, since only two out of 15 participants were men, our findings might not reflect the full range of user experience of men. Third, most participants were educated and computer-savvy; hence the results may not be generalizable to people who are less educated or computer-savvy. Future studies including these populations will be important as they may be in greater need of learning about options, risks and benefits, and exploring their own preferences and engaging in shared decision-making. Fourth, we were unable to address all usability issues identified by participants since some components were already finalized at the time of the testing (e.g., the animated videos). Our choice of animation style was based on a balance between aesthetic and budgetary constraints. Although some participants responded positively to the animated graphic novel approach, others considered it lacking sophistication. Finally, due to the small sample size, we were unable to further explore the influence of demographic characteristics (e.g., age, sex, education level), disease characteristics (e.g., disease duration and severity) and individuals’ internet use (e.g., time spent on internet per day) on the usability scores. This is important because some of the preferences (e.g., animations) may be associated with specific patient characteristics, which if known, would assist in designing future decision aids targeted to particular populations.

Despite the limitations, findings from the usability testing have allowed us to refine the ANSWER prototype. Recognizing that usability issues are major barriers to the adoption of health information technology [42], we have taken steps to address them over the course of the ANSWER’s development. Yen and Bakken recommend three levels of usability evaluation [43]. The first level aims to identify product components and functions needed by users to accomplish a task (i.e., user-task interaction). Methodology includes direct observation and needs assessment using qualitative or survey methodology. The second level assesses the user-task-program interaction using methods such as heuristic evaluation [44], cognitive walkthrough [45], and the think aloud technique [46,47]. The third level examines the complex interaction among users, tasks, the program and the environment using a variety of experimental and observational designs. All three levels are addressed in the ANSWER development and were shown to be helpful for different aspects of refining the tool.

Strengths of the study include the emphasis on user experiences. The ANSWER tool was informed by our previous qualitative research on RA patients’ help-seeking experience, especially their challenges in making medication decisions [29]. In addition, patient/consumer collaborators were involved at the outset to provide input on the program design. We subsequently evaluated the user-task-program interaction in the current usability testing and addressed all modifiable navigation issues. The next step will be to evaluate the ANSWER in a proof-of-concept field study with patients who are considering methotrexate for treating RA. In addition to using the tool online, individuals will be able print the one-page summary of their questions, concerns and preferred option to bring to their rheumatologist appointment. As such, they will have the full experience of shared decision-making. Our goal will be to assess the extent to which the ANSWER reduces decisional conflict and improves self-management knowledge and skills in patients who are considering methotrexate for RA [16].

## Conclusions

We have developed a user-friendly online decision aid to assist patients in making informed decision about using methotrexate for RA. Although the SUS score indicated high usability before and after major modification, findings from the think-aloud sessions illustrated areas that required further refinement. Our results highlight the importance of formative evaluation in usability testing.

## Competing interests

The authors declare that they have no competing interests.

## Authors’ contributions

The study was conceived by LCL, PMA, AFT and CLB. All authors contributed to the development of the research protocol. LCL is the principal applicant and PMA is the decision-maker co-principal applicant. Writing of the manuscript was led by LCL and all authors approved the final version.

## Pre-publication history

The pre-publication history for this paper can be accessed here:

http://www.biomedcentral.com/1472-6947/13/131/prepub
